# Analysis of Epidemiological Characteristics of New Cardiovascular Diseases in Cancer Patients with Cardiovascular Disease

**DOI:** 10.1155/2022/5157398

**Published:** 2022-08-31

**Authors:** Guangchong Zhang, Yiwen Zhang, Wenguan Li

**Affiliations:** ^1^School of Nursing, Henan Medical College, Zhengzhou 451191, Henan, China; ^2^College of Traditional Chinese Medicine, Shandong University of Traditional Chinese Medicine, Jinan, Shandong, China

## Abstract

In cancer patients, a cardiovascular disease (CVD) is a prevalent occurrence. When a patient has both heart disease and cancer, the treatment can be complicated because treatment for one condition can have an adverse effect on the outcome of the other. A cardiovascular disease that involves heart failures, coronary artery disease (CAD), stroke, pericardial diseases, arrhythmias, and valve and vascular dysfunction is a serious worry for long-term cancer patients. Because preclinical research is limited, it is critical to comprehend the pathophysiology of CVD as a consequence of anticancerous therapies while taking into account the developing and expanding heart. As a result, in this research, we look at the epidemiological characteristics of cancer patients who also have cardiovascular illness. Low-dose chest computed tomography, cardiac CT, and cardiac magnetic resonance imaging (MRI) are used to acquire the data and perform the screening. Chemotherapeutic drugs such as anthracyclines and trastuzumab are used to treat the condition. Univariate analysis is used to examine risk factors and predict cardiovascular damage. Sensitivity, specificity, positive predictive value, negative predictive value, life expectancy, left ventricular ejection fraction (LVEF), and longitudinal strain are among the metrics examined.

## 1. Introduction

Cancer is among the world's second major reason for mortality. Improvements in earlier detection, accurate staging, and therapy have led to a considerable minimization in cancer patient death rates and an increase in life expectancy. In the United States, a calculated 14.5 million individuals have a record of cancer. Over the next ten years, this value is expected to climb to 19 million [1]. In the United States, around 12,500 novel instances of cancer in infants and adults are diagnosed each year, with over 300,000 childhood cancer survivors. Long-term deleterious impacts of cancer treatment affect a large percentage of cancer survivors, affecting numerous organ systems. Cancer therapy's cardiovascular damage is a serious worry in this area. Anthracyclines, trastuzumab, and tyrosine kinase inhibitors (TKIs) are among the anticancer drugs linked to an enhanced risk of cardiovascular death and fatality [2]. Survivors of childhood cancer have a substantial lifetime danger of developing late CVD [2]. Left ventricular (LV) dysfunctioning, congestive heart failures (CHF), and pulmonary hypertension are only a few of the cardiovascular consequences of cancer therapy. Cardiotoxicity is more likely in those who have a history of cardiovascular disease. Cardio-oncology is a unique subgroup that concentrates on the complicated interactions between cancer and the cardiovascular systems, as well as the surveillance, earlier identification, prevention, and treatments of cardiotoxicity from cancer therapies, as well as the advancement of novel treatments with reduced or no cardiotoxicity, and cautious outlining of cancer therapies in individuals with pre-existing CVD to prevent observable cardiotoxicity and heart failures [3].

Cancer can have a variety of effects on the cardiovascular system. Primary cardiac tumours are uncommon, although they are frequently related to sensational and fatal medical manifestations. Cancers of the lungs, breast, esophagus, and other mediastinal organs, as well as those that have metastasized to the lungs, shall immediately enter or constrict the cardiac system, major arteries, and pericardial space. Plasma cell malignancies can cause immunoglobulin or its segments to deposit in many organs, involving the cardiac system, causing myocardial dysfunctioning, cardiac failures, and arrhythmias. Hypercoagulability and thromboembolic diseases can be caused by cancers that alter the coagulation cascade. All of these factors are crucial in the field of cardio-oncology [4].

Patients with malignancies have a better prognosis, thanks to advancements in treatment approaches like radiation and systemic treatments [5]. The choice of which anticancer medication to take is influenced by a variety of variables, such as the type and location of the cancer, its severity, whether surgery or radiation therapy can be performed or should be, and the adverse effects of the medication. However, certain anticancer medications can be taken orally, while others can be injected intramuscularly or intrathecally. The majority of anticancer medications are provided intravenously (within the spinal cord). They may, unfortunately, have long-term consequences, such as an elevated risk of CVD in long-term patients [6]. CVDs are important reasons of fatality [7] and death in the general population, accounting for 30–50% of all fatalities in many of the advanced nations. Due to this higher baseline rate, even a slight maximization in CVD risk shall possess a significant effect on fatality and death rates. Following cancer therapy, cardiac diseases can occur as a consequence of direct cardiovascular damages induced by the therapy or as a result of increased atherosclerosis caused by cancer therapy-associated cardiovascular risk elements.

Specificity of anticancer medications is crucial in lowering the degree of adverse effects associated with their use [8]. Because cancer cells are identical to normal human cells, anticancer drugs are typically harmful to normal cells and can produce severe side effects, some of which can be fatal. These adverse effects include hair loss, mouth and mucous membrane ulcers, cardiac abnormalities, bone marrow toxicity, and severe nausea and vomiting. The toxicities of bone marrow result in anaemia and poor resistance to infectious pathogens. Additionally, permanent infertility may develop. These adverse effects may necessitate lowering the medicine's dosage or altering the patient's dosing schedule in order to make the treatment more acceptable.

Streptokinase is a fibrinolytic medication that is manufactured by streptococcal bacteria. Systemically administered streptokinase lyses acute deep-vein, pulmonary, and arterial thrombi; however, it is less efficient in treating chronic occlusions (blockages). When delivered intravenously shortly after a coronary blockage has formed, streptokinase is useful in restoring blood flow through the heart and vessels and reducing the size of the infarct area (tissue death). Streptokinase can also be given directly into coronary blood arteries to deliver a large dose to the clot site. To avoid the recurrence of occlusive clots, heparin, aspirin, dipyridamole, or a combination of these three medications may be added to the treatment plan. A streptokinase overdose may result in bleeding due to systemic fibrinogenolysis, the destruction of coagulation factors by plasmin. Urokinase, a protease enzyme that directly activates plasminogen, is derived from human kidney cells cultured in tissue culture. Urokinase lyses newly produced pulmonary emboli and, unlike streptokinase, it induces fibrinolysis without substantial destruction of coagulation components.

Tissue plasminogen activator (t-PA) promotes fibrinolysis and offers several significant advantages over streptokinase and urokinase for the treatment of coronary thrombosis. It quickly binds to fibrin and, after intravenous administration, activates only the plasminogen that is linked to the clot; hence, fibrinolysis occurs without a broad breakdown of the coagulation components. It may be administered to heart attack patients during route to the hospital, so avoiding the time spent in the hospital prepping the patient for intracoronary streptokinase injections. This is highly valuable since the quick restoration of coronary blood flow is crucial for minimising the extent of cardiac damage following a heart attack.

**Figure 1:**
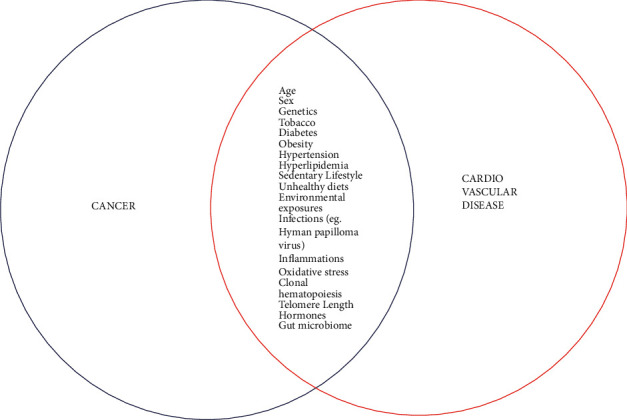
Pathophysiological relation between cancer and CVD.

Long-term cancer survivorship has continued to climb as cancer medicines have evolved, resulting in substantial advancements in the survival rates of a variety of tumours [9]. The possibility of a mechanistic overlap in the frequency of cancer and CVD is becoming more widely recognized [10]. Heart disease and cancer, despite being considered of as two independent disease entities, contribute to half of overall fatalities in the US. Several classic CVD risk factors and underlying pathophysiological mechanisms have been linked to an elevated risk of some malignancies (Figure 1).

Tobacco smoke, which has been directly associated to CVD ever since 1950s and contains well-known toxins connected to lungs, bladder, neck and head, gastro esophageal, pancreas, gastrointestinal, hepatic, and genital tumors, and also leukemia, comes out as a significant modifiable risk factor. Obesity increases the risk for CVD that is irrespective of other risk factors like hypertension, dyslipidemia, and diabetes mellitus. Breast, colorectal, endometrial, gastroesophageal, and severe prostate cancers are all connected to obesity [11]. Inadequate regular exercise and diet rich in saturated fats and poor in fruits and veggies were linked to both disease states [8]. Inflammatory process, oxidative stress, modified telomere length, the gut microbiota, and ambiguous clonal hematopoiesis have all been associated to the development of hematologic tumors, as well as increased atherosclerosis and an elevated risk of coronary heart disease [12].

The link between cardiovascular illness and cancer is thought to be due to common cardiovascular risk elements like becoming aged, smoking, overweight, higher blood pressure, diabetes, hyperlipidemia, and physical inactivities. Short- and long-term heart fatality can both be increased by such mutual risk elements and heart morbidity [13]. Inflammation is an important element in the etiology and advancement of tumor and cardiovascular disorders [14], in addition to conventional risk factors. Tumor therapy has enhanced the overlap between such diseases due to direct cardiotoxic impacts of radiotherapies, chemotherapies, and hormonal cancer treatments, which have been reported to aggravate coronary artery disease when combined with reduced physical activity and weight gain.

Several cancer survivors may have preclinical or medically apparent heart illness at the time of their assessment, which might worsen during therapy [15]. Cardiotoxicity shall restrict cancer therapy, causing it to be interrupted or stopped, and possibly worsening entire results. A “cardiotoxicity prediction” method should be used, which includes monitoring cardiac illness and risk factors, recognizing poor oncological results in the presence of cardiac diseases and risk elements, and changing cancer treatment to one with lesser cardiotoxic profiles. Through the integrated attempts of both cardiology and oncological groups, an ideal tumor survivorship treatment strategy should involve promoting lifestyle changes [16].

The further portion of this article is structured as follows: The problem statement is presented in Section 2. The approach for the analysis is explained in Section 3. The performance of the analysis is examined in Section 4. Finally, Section 5 brings the paper's overarching theme to a close.

## 2. Problem Statement

Despite substantial progress in the prevention, testing, and therapy of diseases, CVD and cancer remain the two major reasons of mortality in affluent nations. Anthracyclines and trastuzumab are key agents in the management of patients with breast cancer. Anthracycline–trastuzumab-containing regimens demonstrate a significant clinical activity in human epidermal growth factor receptor 2 (HER2) positive breast cancer. They are still major public health issues that are becoming increasingly important over the world [17]. Despite this danger, cardiology and oncology have remained mostly separate professions. Because of many associated adaptable risk elements, cancers and CVD frequently overlap in the similar persons; persons identified with lung tumor, breast tumor, or colorectal tumor are at increased risks of CVD, and those with CVD are at greater risks of several kinds of general tumors [18]. Although many testing methods have emerged, there are possibilities to combine cancers and CVD risk evaluation. Integrating cardiovascular and hemato-oncological preventative and research projects may virtually probably result in worldwide public health advantages that are complementary.

## 3. Materials and Methods

**Figure 2:**
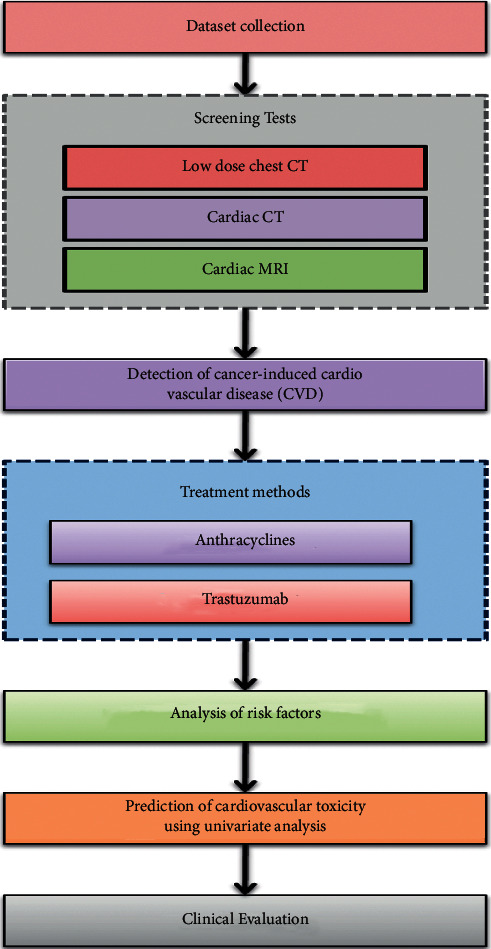
Flow of the methodology used.

The methodology used for the analysis of epidemiological characteristics of patients with cancer combined with cardiovascular disease is discussed in this section. The schematic representation of the suggested methodology is depicted in Figure 2. This experiment was carried out after being approved by the ethics committee of Henan Medical College.

### 3.1. Dataset Collection

The Eindhoven Cancer Registry gathers information on every newly identified tumor patients in China. There are 2.3 million people living in this area, as well as 10 general hospitals and two radiation facilities. The registration is more than 95 percent complete. Patients' features, diagnosis, morphology, histopathology, phase, and commencing therapeutic data are proactively collected from hospital records by experienced registration assistants. The hospital records are typically considered to be the most thorough means of communication regarding a person's historical and contemporary medical status. The Eindhoven Cancer Registry has been collecting significant comorbidity at the time of tumor assessment since 1993. Comorbidity is recorded using a little improved form of the Charlson comorbidity index. Patients who were 50 years or older and had tumor of the colorectal, rectal, non-small-cell lung cancer (NSCLC), small cell lung cancer (SCLC), breast, or prostate (most prevalent tumor forms) between 1995 and 2006 were included. These individuals were included from 2002 to 2006 since preoperative radiation for rectal tumor was only suggested in therapy rules since 2002. Individuals were excluded if an autopsy revealed a cancer diagnosis that did not involve a primary cancer. The predominance and typical features of the tumours were determined at all stages. By stage, the impact of cardiovascular disease on therapy and overall survival was examined.

### 3.2. Screening Tests

European and US recommendations recommend identification of tumor with a lower-dose computed tomography scanning in people who are considered at greater risks based on age, duration of smoking tobacco, and period following discontinuation of smoking amongst ex-smokers. Lung cancer test is done with multidetector CT scanning since the whole chest is examined in a single breathing cycle, subjecting the individual to just 1.5 mSv of irradiation (compared to 8 mSv for a standard CT scan). Low-dose lung CTs may observe and measure CAC values that can forecast CVD occurrences and death, despite the fact that they are not ECG-gated like heart CT. As a consequence, integrating the assessment of both pulmonary parenchyma and CAC as portion of the same CT examination in people who are getting a lung cancer screening may be another way to evaluate tumours and CVD risks. It might provide vital extra details with no need for extra testing or much greater exposure to radiations. Cardiovascular MRI was used in juvenile cancer victims to enhance functional characterization and identify adverse LV remodeling, such as fibrosis, though, as with strain, the long-term diagnostic utility of these abnormalities in this cohort is still to be established. Cardiac magnetic resonance imaging (MRI) might provide testing choices since it permits tissues' characterization and can identify diffused interstitial fibrosis and alterations in localized myocardial functions.

### 3.3. Treatment Methods

#### 3.3.1. Anthracycline-Based Chemotherapy

Anthracycline antibiotic derived from the soil bacterium *Streptomyces peucetius* is highly efficient, wider-spectrum anticancerous drug. Cardiotoxicity is a major disadvantage of its utilization. Such compounds comprise an anthraquinone chain that produces hydrogen peroxide and reactive oxygen species as a consequence of redox cycling. By hybridising among base pairs, anthracyclines limit DNA and RNA creation and hinder DNA damage by inhibiting topoisomerase II activities. Free radical oxidation damages, peroxidation of membrane phospholipids, aberrant calcium management, and reduced fabrication of proteins all contribute to anthracycline cardiotoxicity. Anthracyclines cause myocellular injuries, myocyte failure because of necrosis and apoptosis, left ventricular enlargement, detrimental reconfiguration, reduced distensibility, and congestive heart failures (CHF). Anthracycline cardiotoxicity is split into two kinds: acute and chronic. Acute cardiotoxicity happens right after or immediately after starting the treatment. It is generally short and self-restricting, with a myopericarditis-like look, nonspecific repolarization variations on ECG, dysrhythmias, troponin rise, and acute left ventricle failure. Such aberrations generally resolve on their own with just adjunctive treatment. Persistent anthracycline toxicities, on the contrary, is the most prevalent and serious type of anthracycline toxicities. It comprises left ventricular systolic collapse that is asymptomatic at first but shall develop to dilated cardiomyopathy and observable CHF that are both usually not reversible. Type 1 cardiotoxicity and type 2 cardiotoxicity (often detected after one year of chemotherapy finalization) are arbitrarily defined. The majority of individuals experience chronic cardiotoxicity within the first year of medication. The entire lifespan cumulative dosage of anthracycline is the most critical determinant in anthracycline cardiotoxicity. Higher-intensity chemotherapies might cause cytotoxic effects as an adverse reaction. Higher dose cyclophosphamide treatment, a substantially greater medication, and considerably higher anthracycline dosages enhance the likelihood of having anthracycline cardiotoxicity. Anthracycline cardiotoxicity is hypothesized to be increased by high blood pressure, female gender, previously existing cardiovascular problems, concurrent mediastinal radiation exposure or medication with other cardiotoxic compounds, subclinical iron overload from recurrent blood donations, therapeutic iron supplementations, and iron-handling genetic polymorphisms.

#### 3.3.2. Trastuzumab-Based Chemotherapy

Trastuzumab (Herceptin) is a humanized monoclonal antibiotic, which focuses the HER2/neu proteins, which is deposited on the cellular membranes. Individuals with HER2/neu upregulating breast tumors receive this as a weekly intravenous infusion for up to one year. The HER2 receptors are members of the HER class of receptors. The four categories of HER receptors include HER1 receptor, HER2 receptor, HER3 receptor, and HER4 receptor. Such receptors have extracellular, transmembrane, and intracellular areas. Ligand binding promotes receptor dimerization and stimulation, and also downstream signaling via the PI3K/AKT/mTOR/Ras/MAPK pathway. Cell growth, multiplication, and survivability are all controlled by such mechanisms. Whenever these systems are disrupted, apoptosis and death of cells are induced. HER2/neu is upregulated to 20–25% in tumor tissues. Trastuzumab, particularly combination with other therapies, is effective in HER2-overexpressed tumor tissues. Trastuzumab can cause cytotoxic effects as a secondary effect. Nevertheless, unlike anthracyclines, its toxicities and cardiac dysfunctions have a different vision, method, and disease symptoms. Trastuzumab induces LV malfunction and CHF, both of which could be reversed if the treatment is stopped. Trastuzumab, unlike anthracyclines, promotes cardiac dysfunctions that are unrelated to cumulative dosage and irrelevant to the ultrastructural changes in cardiomyocytes found with anthracyclines. Moreover, the risk elements for developing trastuzumab-related LV dysfunctions, aside from age >65 years and simultaneous or previous anthracycline treatment, remain unknown. Myocytes have HER2 receptor on their surfaces, which perform a function in cardio protection and angiogenesis. This explains why trastuzumab, a drug that blocks HER2 receptor activity, is cardiotoxic.

### 3.4. Analysis of Risk Factors

Chemotherapeutic treatment is usually aggravated by cardiovascular disorders also including heart failures, myocardial infarctions, high blood pressure, thrombosis, QT lengthening, and bradycardia. Cardiotoxicity has been linked to a number of different chemotherapy categories and therapeutic strengths. Every trial's medication dosage, the accumulated dosages, the manner of injection, the mixture of medications supplied, the interaction with radiation, and the distribution intervals are all elements that leads to the growth of cardiotoxicity. The person's age, existing cardiovascular risk elements, and/or diseases all contribute to cardiotoxicity. Antimetabolites, antimicrotubule compounds, monoclonal antibody-dependent tyrosine kinase inhibitors, and monoclonal antibody-dependent smaller molecule tyrosine kinase inhibitors are all examples of anthracyclines. Anthracycline-induced cardiotoxicity was already thoroughly studied. Anthracycline-mediated cytotoxicity is thought to be caused by the production of reactive oxygen species (ROS) and the formation of iron compounds, which together result in intrinsic damages. As per this study, topoisomerase II (Top2) is needed for anthracycline to cause DNA double-strand breakage and transcriptome changes, leading in mitochondrial dysfunctioning and the ROS. Anthracyclines promote dose-associated cardiac cell damages and also persistent disruption of reparatory and homoeostatic processes following treatment.

A further suggested scheme of chemotherapy-induced cardiotoxicity in people with cancer is aberrant vasoreactivity induced by endothelial dysfunction and variations in the regulation of vascular smooth muscle strength [19]. Anthracyclines and trastuzumab have all been linked to myocardial injury in persons who have or have not had a heart attack. Microvascular injury, in addition to coronary vasospasm, is yet another cardiotoxic process suggested for TKIs including sorafenib that has been related to coronary vasospasm in many arteries [20]. As a consequence of endothelial dysfunctioning, inflammatory processes, platelet aggregation, and vascular reconfiguration, chemotherapeutic compounds that produce severe coronary thromboembolism, like bevacizumab or cisplatin, may promote myocardial ischemia [21]. Some of the negative effects related with cancer therapy are included in Table 1.

### 3.5. Prediction of Cardiovascular Toxicity Using Univariate Analysis

Cardiovascular illness and risk elements are significant forecasters of tumor-related cardiotoxicity. In patients getting anthracycline chemotherapy, CAD, higher blood pressures, and diabetes are amongst the potential forecasters of left ventricular dysfunctioning, whereas in breast tumor individuals obtaining trastuzumab, CAD, higher blood pressures, and obesity enhance the risks of LV dysfunctions. In individuals obtaining antiangiogenic targeted therapies for a range of malignancies, previously existing hypertension is the single highest forecaster of acute hypertension necessitating suspension of tumor treatments. In individuals obtaining anthracyclines or trastuzumab for gastrointestinal tumors, coronary artery disease raises the risks of coronary artery vasospasm. Unregulated heart disease or CVD risk elements may contribute towards less severe cancer treatment and poorer cancer results because any of these adverse effects may impair the capacity to give comprehensive cancer treatments.

Physicians may modify preventive treatments and strengthen cardiac surveillance to minimize the abrupt and long-term impacts of cancer treatment on cardiovascular health if they can categorize patients at increased risk for cardiotoxicity before initiating treatment for cancer. Correct assessment of people of lower risk of cardiovascular issues should decrease the necessity for unnecessary cardiac monitoring in this group, improving patients' quality of life and freeing up healthcare resources for those at greater risk. Cardiotoxicity risk from cancer therapy has been predicted using univariate analysis including patient-specific characteristics to create personalized cardiac risk scores.

## 4. Results and Discussion

The parameters like sensitivity, specificity, positive predictive value, negative predictive value, life expectancy, left ventricular ejection fraction (LVEF), and longitudinal strain are all examined in this section.

### 4.1. Life Expectancies Free of Cancer and Cardiovascular Diseases

**Figure 3:**
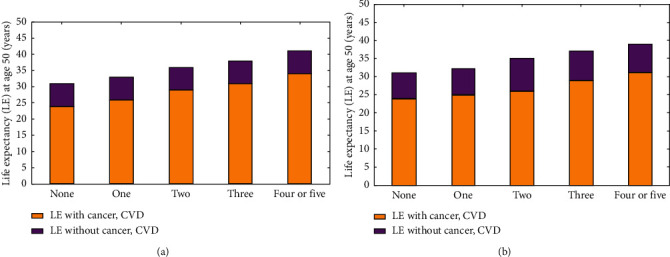
Deaths from cancer and cardiovascular disease (CVD) among individuals aged 50 years and older. (a) Study of nurses' health (women). (b) Follow-up study (men) based on the count of lifestyle elements that are considered to be at low risk.

Overall life expectancy at 50 years increased as the count of lower-risk lifestyle elements grew: women's life expectancy increased from 31.7 to 41.1 years, and men's life expectancy climbed from 31.3 to 39.4 years. The broad mass of life expectancy beyond 50 years was free of tumor and cardiovascular diseases, irrespective of the count of lower-risk lifestyle elements (Figures 3(a) and 3(b)). Cigarette smoking, physical activities, higher diet qualities, medium alcohol absorption of 5–15 g/day (women) or 5–30 g/day (men), and normal weight (BMI of 25) were all low risk lifestyle factors. Morbidity and fatality rates for lower-risk lifestyle variables contrasted to zero lower-risk lifestyle elements (sex-specific) adapted for age and identity, present multivitamin utilization, present aspirin utilization and family history of diabetes or myocardial tumor and menopausal hormone utilization (women only).

Women who embraced 0, 1, 2, 3, and 4 or 5 lower-risk lifestyle elements at the age of 50 had life expectancies of 23.7, 26.4, 29.1 and 31.8 years, respectively, free of cancer and cardiovascular disease. Life expectancy free of cancer and CVD at age 50 among males who embraced 0, 1, 2, 3, and 4 or 5 lower-risk lifestyle elements was 23.5, 24.8, 26.7, 28.4, and 31.1 years, respectively (Table 2). 74.8 percent, 76.6 percent, 80.1 percent, 82.2 percent and 83.6 percent of total life expectancies were free of cancer and CVD among females who had embraced zero, 1, 2, 3, or 4 or 5 lower-risk lifestyle variables, accordingly.

A rise in life expectancy free of cancer and cardiovascular disease after the age of 50 was found to be connected with growing numbers of lower-risk lifestyle elements (Figure 3(b)). Females and men with 4 or 5 lower-risk lifestyle elements lived an average of 10.3 and 7.2 years longer than those with 0 lower-risk lifestyle elements in our sensitivity assessment that stopped upgrading lifestyle elements following an identification of tumor and cardiovascular diseases.

### 4.2. Life Expectancy in Existence of Cancer and CVD

Cancer and cardiovascular diseases were all related to a lower life expectancy in the low-risk group, which may be attributable to a lesser count of individuals in that class as well as a greater time to live following assessment. Women had a hazard ratio of 0.5, while men had a hazard ratio of 0.6. This means that those who had 4 or 5 lower-risk lifestyle elements were far less likely to develop cancer, or cardiovascular disease, than those who had none of these factors. Following identification of such diseases, half of individuals with tumor who embraced 4 or 5 lower-risk lifestyle elements remained up to 23.3 years, whilst half of individuals with tumor who embraced 0 lower-risk lifestyle elements remained only up to 12.3 years. More than two-thirds of women and two-thirds of men were found to be at risk for tumor or CVD if they had no lower-risk lifestyle elements (no risk elements, one risk element, two risk elements, three risks elements, and 4 or 5 risk elements), accordingly.

More than 10 years higher life expectancy without severe chronic diseases was seen in females who had 4 or 5 lower-risk lifestyle variables in their favor, compared to women whose lives were otherwise unaffected by these conditions. Amongst females, 4 to 5 lower-risk lifestyle characteristics were related with an additional 8.3 years of cancer-free life expectancy and an additional 10 years of cardiovascular disease-free life expectancy (Table 2). Men with 4 to 5 lower-risk lifestyle elements had a 8.5 year greater life expectancy without vast chronic diseases, including 6.0 more years without cancer, and 8.7 additional years without CVD, contrasted to those with 0 lower-risk lifestyle elements (Table 2).

### 4.3. Cardiotoxicity

Anthracycline-induced cardiotoxicity was discovered in one patient at the 3-month mark, whereas the onset of cardiotoxicity was delayed in another eight patients for a period of six months. Echocardiographic and blood markers were not significantly different between those who developed cardiotoxicity and those who did not, at baseline. There was no correlation between the variation in the LVEF from baseline to 3 months (*p*=0.19; Table 3) and later cardiotoxicity. As this was also the case, individuals were split into two categories depending on whether or not their LVEF decreased by 5 percent.

Patients who experienced cardiotoxicity after three months had decreased longitudinal and radial strains and raised hsTnI, whereas the reverse was true (Table 3). Individuals who did not possess cardiotoxicity had fewer anomalous segments at 3 months than individuals who did. Cardiotoxicity was predicted by the count of aberrant segments at 3 months (*p*=0.01 for both criteria). There was no correlation between the reduction in circumferential strain and the development of cardiotoxicity. Cardiotoxicity could not be anticipated based on changes in NT-proBNP levels over the course of three months or on an NT-proBNP level above the usual range. Cardiotoxicity at 6 months was not predicted by any of the echocardiographic indicators of diastolic functions at 3 months or their variation from 0 to 3. Increased high‐sensitivity troponin I (hsTnI) at 3 months (*p*=0.02) and a reduction in longitudinal strain between baseline and 3 months (*p*=0.02) stayed independent forecasters of subsequent cardiotoxicity, according to multiple logistic regression.

### 4.4. Parameters of the Forecasters of Cardiotoxicity

**Figure 4:**
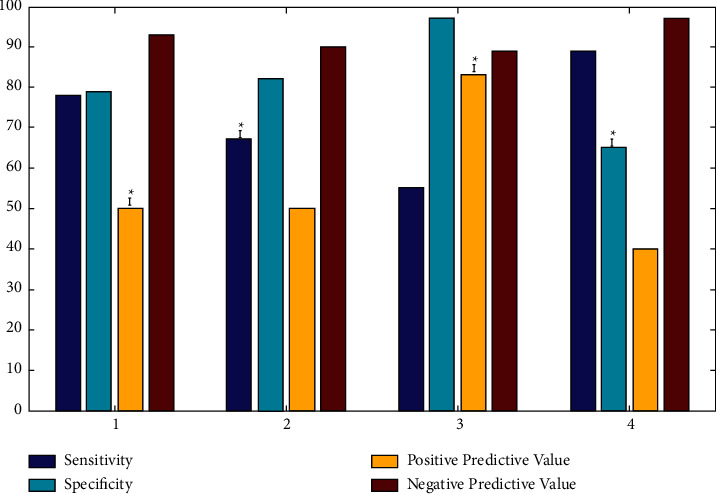
Parameters of the predictors of cardiotoxicity.

Between 0 and 3 months, longitudinal strain reduced by 10% in 14 individuals (32 percent). Twelve patients had high HsTnI levels at three months (28 percent).  Table 4 and Figure 4 show the sensitivity, specificity, and positive predictive value and negative predictive value of hsTnI advancement and a 10% reduction in longitudinal strain.

### 4.5. Cardiovascular Deaths during Follow-Up

**Figure 5:**
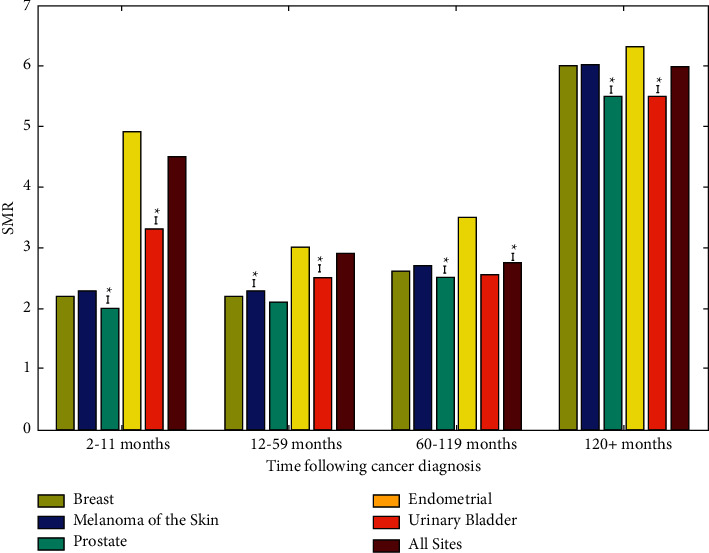
2000–2015 risk of death from heart disease.

For the purpose of this study, we looked at cancer locations where patients had a 30 percent chance of death from the malignancy and a 20 percent risk of death from cardiovascular disease (CVD).  Figure 5 shows that the endometrial tumor had the highest risk of fatality from heart diseases at every time point following diagnosis. Our findings also showed a greater risk of heart disease mortality among individuals with cancers such as breast, melanoma, and prostate, compared to the first year following their diagnosis.

## 5. Conclusions

Although much has already been learned about cancer therapy-associated cardiotoxicity in the past, there is still much more that may be performed, even as clinical practice evolves and the resulting information must be taken with caution. The late impacts of contemporary systemic therapies and radiotherapies on important heart morphologies, as well as possible interactions between treatment modalities, require more research. As a result, in this paper, we looked at the epidemiological characteristics of cancer patients who also had cardiovascular illness. The univariate analytic method was used to predict cardiovascular toxicity. The existence of CVD in cancer patients has been found to influence treatment and treatment outcome in this study. This understanding will assist cancer individuals live longer and have a finer quality of life, with lesser therapy-associated fatality from other diseases.

## Figures and Tables

**Table 1:** Some systemic cancer therapies having cardiovascular negative impacts [22].

Cancer therapies	Cardiovascular effects	Long-term effects	Mechanisms
Anthracyclines	Cardiotoxicity type I irreversible	Present	Losses of myocardium
Cyclophosphamide		Unlikely	Myocarditis
Cisplatin		Unlikely	Unrecognized
Pyrimidine analogues	Myocardial ischemia	Unlikely	Coronary vasospasm
Anti-VEGF therapies		Unlikely	Arterial thrombosis
Arsenic trioxide	Arrhythmia	Absent	HERG *K*+ blockages
Selected TKIs			HERG *K*+ blockages
Cisplatin	Thromboembolism	Unlikely	Endothelial damages
Anti-VEGF therapeutics			Endothelial damages
Selected TKIs	Pleural effusion	Unrecognized	Unrecognized
Chosen TKIs	Peripheral arterial occlusive diseases	Unrecognized	Unrecognized
Selected TKIs	Pulmonary hypertension	Unrecognized	Unrecognized
Anti-VEGF therapies	Arterial hypertension	Unrecognized	Multiple processes
Anti-HER2 therapies	Cardiac dysfunctioning	Unlikely, except when integrated with anthracyclines	Mitochondrial dysfunctioning
Anti-VEGF therapeutics	Type II reversible	Unlikely	Mitochondrial dysfunction

**Table 2:** Life expectancies at 50 years in absence of cancer and CVD as per the count of lower-risk lifestyle elements in nurses' health study (females) and health professionals follow-up study (males).

	No. of low-risk lifestyle factors				
	Zero	One	Two	Three	Four or five
*Females*					
Free of cancer, CVD	24.8 (23.6 to 25.7)	27.5 (26.7 to 27.5)	30.2 (29 to 30.5)	31.9 (30.9 to 33.3)	35.5 (33.2 to 35.6)
Life expectancy difference	Reference	2.6 (2.3 to 2.9)	6.3 (5 to 5.6)	9.1 (7.8 to 8.5)	11.7 (10 to 12.4)
Free of cancer	27.7 (26.5 to 29.8)	30.8 (29.5 to 30.9)	32.8 (31.6 to 32.8)	34.9 (32.8 to 35.9)	36.9 (35.7 to 38.2)
Life expectancy difference	Reference	3.3 (1.9 to 2.5)	5.2 (4 to 4.6)	7.2 (5.9 to 6.7)	9.4 (6.7 to 9)
Free of CVD	31.3 (30.3 to 30.6)	33.7 (33.2 to 33)	36 (34.6 to 36.5)	38.6 (37 to 38.3)	41.2 (40.5 to 41.9)
Life expectancy difference	Reference	2.5 (2.1 to 2.7)	5.7 (4.5 to 5.3)	8.4 (6.9 to 7.8)	11 (9.4 to 10.7)

*Males*					
Free of cancer and CVD	24.5 (23.6 to 24.7)	27.5 (26.7 to 27.5)	27.2 (26 to 27.5)	28.9 (30.9 to 33.3)	35.5 (27.2 to 25.6)
Life expectancy difference	Reference	2.6 (2.3 to 2.9)	3.3 (2.7 to 4.6)	5.1 (6.8 to 6.5)	5.7 (7 to 6.4)
Free of cancer	27.4 (26.8 to 28.8)	30.8 (29.5 to 30.9)	30.8 (28.6 to 31.8)	32.9 (30.8 to 33.9)	36.9 (35.7 to 38.2)
Life expectancy difference	Reference	3.3 (1.9 to 2.5)	2.2 (2.2 to 2.8)	3.2 (4.9 to 4.7)	9.4 (8.6 to 9)
Free of CVD	30.3 (29.3 to 30.6)	31.7 (29.2 to 31.2)	36 (34.6 to 36.5)	38.6 (37 to 38.3)	41.2 (40.5 to 41.9)
Life expectancy difference	Reference	1.5 (2.1 to 1.7)	5.7 (4.5 to 5.3)	8.4 (6.9 to 7.8)	11 (9.4 to 10.7)

**Table 3:** Univariate assessment of predictors of cardiotoxicity.

Variables	Cardiotoxicity		*p* value (forecast of cardiotoxicity)	Odds ratios	95% confidence interval
	Absent (*n* = 34)	Present (*n* = 9)			
Variation in the LVEF at 3 months (%)	1.3 ± 7	6.7 ± 8	0.18	5.6	0.46–100
Variation in longitudinal strain at 3 months (%)	4 ± 11	16 ± 9	0.01	501	6.8–110.000
Variation in radial strain at 3 months (%)	3 ± 24	23 ± 23	0.03	251	4–40.000
Variation in NT-proBNP at 3 months (%)	47 ± 245	57191±	0.92	1	0.65–1.4
Elevation hsTnl at 3 months	7 (18%)	6 (68%)	0.007	9	1.9–50

**Table 4:** Parameters of the forecasters of cardiotoxicity.

Predictors	Sensitivity	Specificity	Positive predictive value	Negative predictive value
(1) 10% decrease in longitudinal strain	7/9 (79%)	27/34 (79%)	7/14 (50%)	27/29 (93%)
(2) Elevated hsTnl at 3 months	6/9 (68%)	29/35 (83%)	6/12 (50%)	28/31 (90%)
(3) 10% decrease in longitudinal strain and elevated hsTnl at 3 months	5/9 (56%)	5/6 (98%)	5/6 (84%)	33/38 (89%)
(4) 10% decrease in longitudinal strain and increased hsTnl at 3 months	8/9 (90%)	22/34 (66%)	8/20 (40%)	22/23 (97%)

## Data Availability

The datasets and supporting data are available from the corresponding author upon reasonable request.
